# Role of Electron Microscopy in the Diagnosis of Cadasil Syndrome: A Study of 32 Patients

**DOI:** 10.1371/journal.pone.0065482

**Published:** 2013-06-17

**Authors:** Manrico Morroni, Daniela Marzioni, Michele Ragno, Paolo Di Bella, Elisabetta Cartechini, Luigi Pianese, Teresa Lorenzi, Mario Castellucci, Marina Scarpelli

**Affiliations:** 1 Department of Experimental and Clinical Medicine, Section of Anatomy, School of Medicine, Università Politecnica delle Marche, Ancona, Italy; 2 Electron Microscopy Unit, United Hospitals, Ancona, Italy; 3 Division of Neurology, Mazzoni Hospital, Azienda Sanitaria Unica Regionale, Zona Territoriale 13, Ascoli Piceno, Italy; 4 Department of Neurosciences, School of Medicine, Università Politecnica delle Marche, United Hospitals, Ancona, Italy; 5 Division of Neurology, Macerata Hospital, Azienda Sanitaria Unica Regionale, Zona Territoriale 9, Macerata, Italy; 6 Molecular Medicine Laboratory, Mazzoni Hospital, Azienda Sanitaria Unica Regionale, Zona Territoriale 13, Ascoli Piceno, Italy; 7 Department of Biomedical Sciences and Public Health, Section of Pathological Anatomy, School of Medicine, Università Politecnica delle Marche, United Hospitals, Ancona, Italy; University of Cambridge, United Kingdom

## Abstract

**Background and Purpose:**

Cerebral autosomal dominant arteriopathy with subcortical infarcts and leukoencephalopathy (CADASIL) is caused by NOTCH3 gene mutations that result in vascular smooth muscle cell (VSMC) degeneration. Its distinctive feature by electron microscopy (EM) is granular osmiophilic material (GOM) detected in VSMC indentations and/or the extracellular space close to VSMCs. Reports of the sensitivity of EM in detecting GOM in biopsies from CADASIL patients are contradictory. We present data from 32 patients clinically suspected to have CADASIL and discuss the role of EM in its diagnosis in this retrospective study.

**Methods:**

Skin, skeletal muscle, kidney and pericardial biopsies were examined by EM; the NOTCH3 gene was screened for mutations. Skin and muscle biopsies from 12 patients without neurological symptoms served as controls.

**Results and Discussion:**

All GOM-positive patients exhibited NOTCH3 mutations and vice versa. This study i) confirms that EM is highly specific and sensitive for CADASIL diagnosis; ii) extends our knowledge of GOM distribution in tissues where it has never been described, e.g. pericardium; iii) documents a novel NOTCH3 mutation in exon 3; and iv) shows that EM analysis is critical to highlight the need for comprehensive NOTCH3 analysis. Our findings also confirm the genetic heterogeneity of CADASIL in a small Italian subpopulation and emphasize the difficulties in designing algorithms for molecular diagnosis.

## Introduction

Cerebral autosomal dominant arteriopathy with subcortical infarcts and leukoencephalopathy (CADASIL) is a genetic disorder caused by mutations in the NOTCH3 gene, which maps to chromosome 19 and encodes the transmembrane receptor NOTCH3 [1], [2]. The postnatal expression of the gene is mostly restricted to vascular smooth muscle cells (VSMCs) of middle and small arteries and pericytes [1], [3]. The mutations are associated with accumulation of the NOTCH3 extracellular domain in the wall of small cerebral arteries, resulting in VSMC degeneration [4], [5], [6].

The disease is characterized by migraine, transient ischemic attacks (TIAs) and/or strokes, cognitive decline and psychiatric symptoms [7]. Typical magnetic resonance imaging (MRI) features are severe leukoencephalopathy frequently involving the temporal pole and the external capsule, lacunar lesions, and microbleeds [8], [9], [10]. Microscopic features are arterial wall fibrosis and thickening and alterations of smooth muscle cells, which eventually disappear. Electron microscopy (EM) shows deposits of granular osmiophilic material (GOM) in VSMC indentations or in the extracellular space in close vicinity to VSMCs [11], [12], [13]. Even though GOM was originally described in the CNS, it is now accepted that CADASIL is a systemic disease [13], [14], [15], [16], [17]; accordingly, GOM has also been identified in skin and muscle biopsies [4], [18], [19]. Over the past 12 years we had the opportunity to examine skin and muscle biopsies from several patients suspected to have CADASIL. In one patient GOM was found in the pericardium, a tissue where it had never been described before. This study presents the findings obtained in a series of 32 patients and discusses the role of EM in CADASIL diagnosis. Our findings highlight the diagnostic value of EM in patients with clinical and MRI findings strongly suggestive of CADASIL but not showing the more common NOTCH3 mutations in earlier analyses and provide additional information on the syndrome.

## Materials and Methods

### Ethics statement

This study was approved by the Università Politecnica delle Marche Research Ethics Committee. Written informed consent was obtained from all participants and is recorded on file. The procedures followed were in accordance with institutional guidelines.

### Patients

The study includes 32 individuals (14 men and 18 women) with a suspected clinical diagnosis of CADASIL; their age ranged from 29 to 74 years (mean 51 years).

The suspicion was based on a family history compatible with an autosomal dominant inheritance, MRI findings suggestive for multiple cerebral infarcts and leukoencephalopathy (involving in particular the temporal pole and/or the external capsule), and clinical findings such as recurrent TIA or stroke, cognitive defects, epilepsy, migraine and psychiatric symptoms.

Samples were collected over a period of 12 years (2001–2012).

Given the existence in the area of a strong Electron Microscopy Unit and the difficulty to obtain a genetic analysis, especially in the earlier years of the study, ultrastructural examination of skin and muscle biopsies was the first approach to the diagnosis. In 27 patients molecular analysis of the NOTCH3 gene was done soon after the EM report; in 4 patients (#1, #2 and #3 belonging to the same family, and #6; see Table 1) it was performed many years after the EM study; and in the last patient (#10; Table 1) it actually preceded it.

**Table 1:** Genetic findings detected in GOM-positive patients suspected to have CADASIL.

*Pt #*	*Gender*	*Age/years*	*Sample*	*GOM*	*Mutation in*	*Amino acid change*
*1	M	51	skeletal muscle	+	exon 3	C108S^a^
*2	M	57	skin	+	exon 3	C108S^a^
*3	F	50	skeletal muscle	+	exon 3	C108S^a^
4	M	45	skin and kidney	+	exon 19	R1006C
5	F	64	skin	+	exon 2	R54C
6	M	47	skin	+	exon 10	G528C
7	M	53	skin	+	exon 10	G528C
8	M	48	skeletal muscle	+	exon 10	G528C
9	F	46	skin	+	exon 3	R110C
10	F	56	pericardium	+	exon 10	G528C
11	M	59	skin	+	exon 4	R141C
12	F	54	skin	+	exon 10	G528C
13	M	54	skin	+	exon 6	R332C

Pt: patient.

* Patients from the same family (siblings).

+ presence of GOM.

a Unpublished mutation.

A punch skin (n = 23) or a muscle biopsy (n = 4) was provided in 27 cases; a pericardial tissue fragment obtained during open heart surgery for valve replacement was available for one patient (#10; Table 1); both skin and muscle biopsies were available for 3 more patients, and a skin and a kidney biopsy were provided for the last (#4; Table 1). Muscle (#1 and #3) or skin (#2) was examined in the 3 siblings (Table 1). Skin (n = 9) and muscle biopsies (n = 3) from 12 patients (6 men and 6 women, age range: 35–75 years, mean 55.3) without neurological symptoms were used as negative controls for EM. They suffered from mycosis fungoides (n = 4), autoimmune disease (n = 4), Stevens-Johnson syndrome, pseudoxanthoma elasticum, leishmaniasis, and Milker's nodule (one case each).

### Electron microscopy

All samples were fixed in 2% glutaraldeyde/2% paraformaldehyde in 0.1 M phosphate buffer, postfixed in buffered osmium tetroxide, dehydrated in graded alcohols, and embedded in an Epon-Araldite mixture. Semithin sections were cut and stained with toluidine blue to select arteries of appropriate size for thin sectioning. Thin sections were stained with lead citrate and examined with a CM10 transmission electron microscope (Philips, Eindhoven, The Netherlands). The sample was considered adequate when it contained at least five arteries with multiple VSMC layers and the inner elastic lamina [20]. More than one thin section per patient often had to be examined to achieve a sufficient number of vessels.

### NOTCH3 gene analysis

Molecular analysis of the NOTCH3 gene was performed according to a previously described protocol [21]. Briefly, genomic DNA was isolated from peripheral blood leukocytes with the MagNA Pure LC DNA isolation kit by an automated nucleic acid extractor (MagNA Pure extractor, Roche, Basel, Switzerland). DNA was amplified by PCR to screen the exons containing EGF-like repeats (exons 2–23) and exons 24 and 25. In the earlier years of the study the diagnostic protocol envisaged the search for mutations only in exons 3 and 4, where most CADASIL mutations have been reported; in 2005 screening was extended to all exons containing EGF-like repeats and to exons 24 and 25. The PCR products were directly sequenced for both sense and antisense strands using an automated fluorescent sequencing method (Big Dye® Terminator v3.1 Cycle Sequencing Kit) (Applied Biosystems, Foster City, CA, USA) on an ABI Prism 3130 Genetic Analyzer (Applied Biosystems) after treatment with exonuclease I and shrimp alkaline phosphatase (ExoSap-IT, USB Corporation, Staufen, Germany). The sequences of the PCR products were aligned with the published human NOTCH3 DNA sequence to identify any sequence changes using the SeqScape software (Applied Biosystems).

## Results

**Figure 1:**
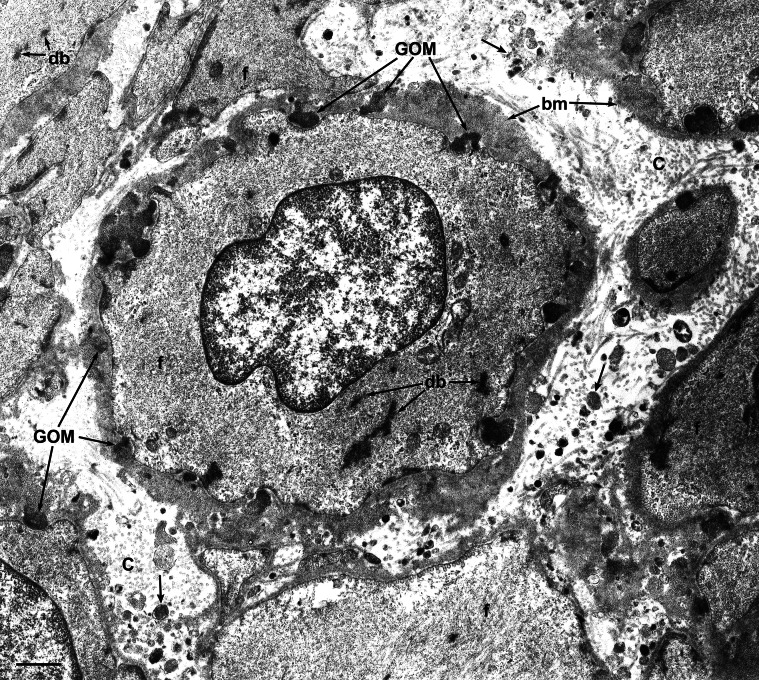
Transmission electron microscopic image of a muscle biopsy from a GOM-positive patient (#1). Smooth muscle cells in the tunica media of a small artery. The cytoplasm, which in one cell includes the nucleus, contains the characteristic organelles of smooth muscle cells: filaments (f) and dense bodies (db). Cells are surrounded by the basal membrane (bm). Several GOM deposits can be seen outside the cells, often in cell membrane invaginations. The small clumps of electron dense material (arrows) between cells and included in collagen (C) are not GOM but debris, probably deriving from cell degeneration. Scale bar = 0.6 µm.

**Figure 2:**
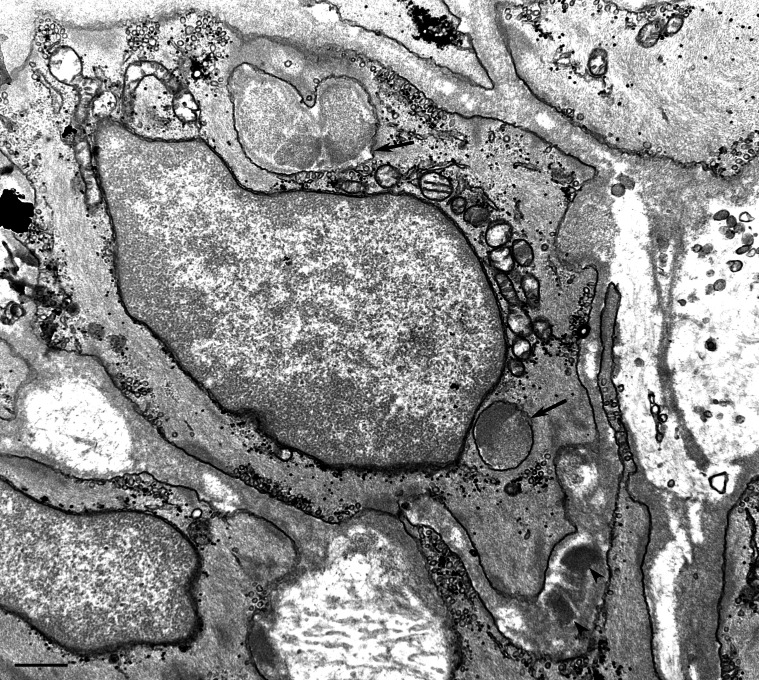
Transmission electron microscopic image of a skin biopsy from a GOM-positive patient (#12). Two GOM deposits located outside the smooth muscle cell membrane (arrowheads). Due to the plane of section and to the irregular indentations, the cytoplasm of a VSMC contains two GOM pseudoinclusions (arrows). Scale bar = 0.5 µm.

**Figure 3:**
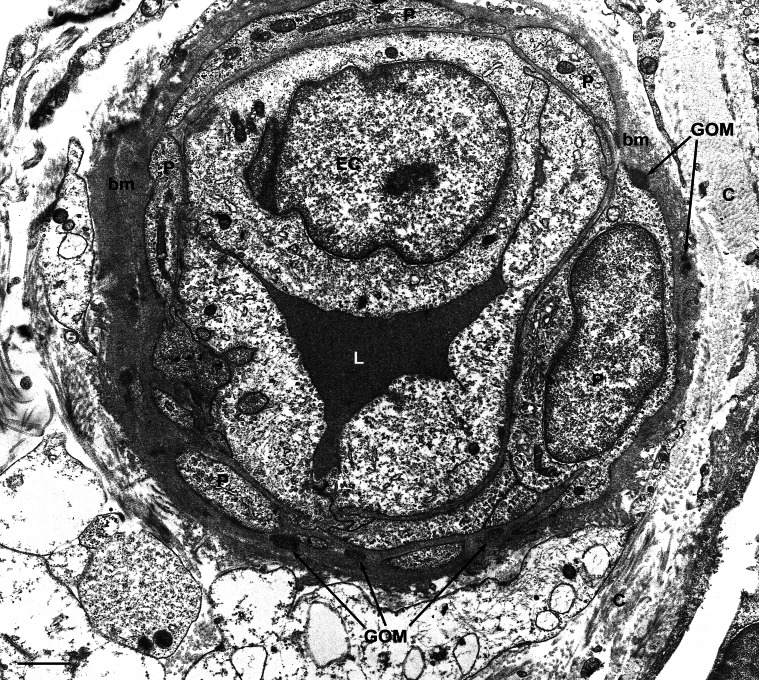
Transmission electron microscopic image of a skin biopsy from a GOM-positive case (#2). This dermal capillary shows a thickened basal membrane (bm) and is surrounded by pericytes (P). Numerous GOM deposits can be seen in the basal membrane and very often in close vicinity to pericytes. C, collagen; EC, endothelial cell; L, capillary lumen. Scale bar = 0.6 µm.

**Figure 4:**
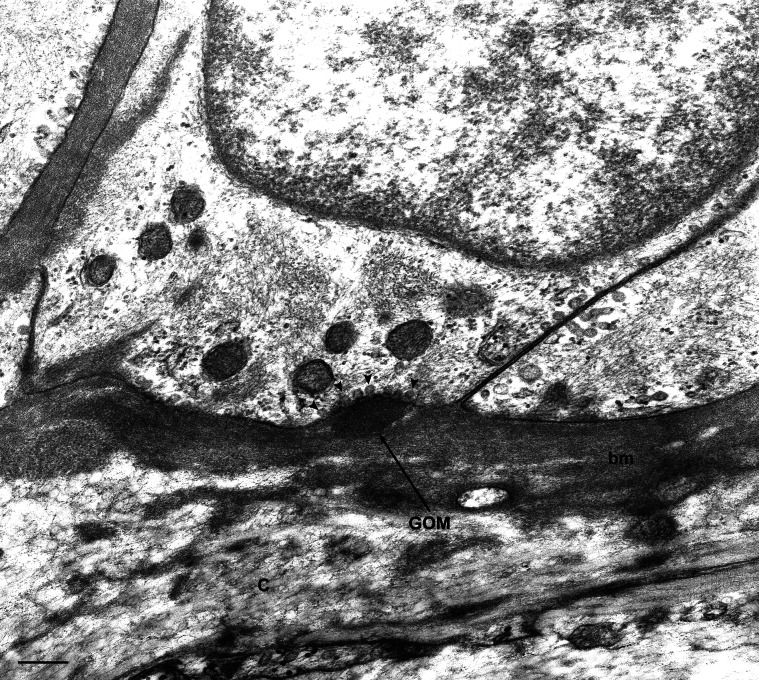
Transmission electron microscopic image of a skin biopsy from a GOM-positive case (#5). GOM located in an infolding of a VSMC. The cell membrane shows numerous pinocytotic vesicles close to the GOM deposit (arrowheads). C, collagen; bm, basal membrane. Scale bar = 0.2 µm.

GOM was found in 13 patients, 8 men and 5 women (including the 3 siblings) aged 45 to 64 years (mean 52.5), who were strongly suspected to have CADASIL. GOM was identified in 9 skin, 3 muscle, 1 kidney and 1 pericardial tissue biopsy (Table 1). There were no differences in GOM location or distribution between the analysed tissues. GOM was found at extracellular sites, most often near VSMC indentations or close to VSMCs of small and medium-sized arteries (Fig. 1). In patient #12 the VSMC indentations were very irregular and formed cytoplasmic pseudoinclusions containing GOM (Fig. 2). Occasionally, GOM was also detected in capillaries, where it was predominantly located very close to pericytes (Fig. 3), and in vein walls (not shown). The indented cytoplasmic membrane of smooth muscle cells and pericytes showed numerous pinocytotic vesicles in proximity of the GOM deposits (Fig. 4). Besides GOM, vessel walls frequently exhibited abnormalities such as smooth muscle cell degeneration and cell loss, thickening of the basal membrane, and abnormal endothelium due to the presence of vacuoles, which have all been described in CADASIL patients. Very few arteries containing GOM exhibited mild wall changes. No distinctive ultrastructural abnormalities were shared by the three siblings (#1, #2 and #3) or set them apart from the other patients, demonstrating that qualitative or semi-quantitative changes in GOM do not correlate with age. In patient #4 (skin+kidney biopsy) the skin sample showed abundant GOM, and molecular analysis disclosed the R1006C CADASIL mutation in exon 19 of the NOTCH3 gene. In the kidney biopsy, obtained at a later time due to renal symptoms, light microscopy showed slight, non-specific glomerular and tubulointerstitial injury and homogeneous thickening of the wall of small arteries. Immunofluorescence microscopy for immune complexes was negative. Moreover EM disclosed GOM in interlobular and juxtaglomerular arteries. This case has already been reported [22].

A NOTCH3 gene mutation was found in all 13 GOM-positive patients (Table 1). However, no genotype-phenotype correlations were found in this group. Genetic analysis of the samples from the three siblings became possible 12 years after the EM study. All three showed a novel mutation in exon 3 of the NOTCH3 gene, a nucleotide substitution c.322T>A involving a pathogenic substitution of a cysteine with a serine at codon 108 (p.C108S). In patient #6 genetic analysis, carried out soon after the skin biopsy, was directed only at exon 3 and 4 mutations and was negative. The EM data prompted further NOTCH3 gene screening (exons 2–24), which documented a mutation in exon 10.

Granular debris, not GOM, was found in the other 19 patients, 6 men and 13 women aged 29 to 74 years (mean 50.2 years). Frequently detected in the inner elastic lamina or near VSMCs in arterial walls, it was organized as small clumps and may conceivably derive from degenerated cells. Granular debris found in a biopsy submitted to confirm the clinical suspicion of CADASIL is a potential source of error. Comprehensive testing for NOTCH3 gene mutations was negative in all these 19 patients; their clinical follow-up was consistent with the EM and genetic findings, supporting the exclusion of CADASIL.

The 12 patients without neurological symptoms, included as controls, were negative for GOM as well as for NOTCH3 mutations.

## Discussion

In all 32 patients biopsies were collected as part of the initial diagnostic work-up due to the difficulty in obtaining genetic analysis, especially in the earlier years of the study. At our integrated University-Hospital institution EM examination has been performed for more than 35 years to support a variety of diagnoses including skin, kidney and muscle diseases, an approach that has helped to create a remarkable expertise in these fields of pathology.

GOM was found in all tissue samples from our patients, i.e. skin, muscle, kidney and pericardium, confirming that CADASIL is a systemic vascular syndrome and that it may exceptionally be associated with renal disease [13], [22], [23], [24], [25].

Identification of GOM in three siblings led to extensive genetic testing and identification of a previously unreported mutation, which adds to the complexity of the NOTCH3 gene variation database [26].

Our data confirm the high sensitivity of EM for CADASIL diagnosis described in previous studies [26], [27], [28], [29], [30]. In particular, they agree with the results reported by Tikka et al. [26], who identified GOM in all their 131 mutation-positive cases. Even though the present study involved a much smaller sample, it is worth noting that all patients but one were from a small area in central Italy (population about 700,000), whereas the 131 patients of Tikka et al. [26] came from an area encompassing Finland (n = 38), Sweden (n = 13) and France (n = 80).

Confirmation of the view of Mayer et al. [28] that skin biopsies are as reliable as muscle biopsies for CADASIL diagnosis was an additional finding. Moreover, we detected GOM in the pericardium; to the best of our knowledge this is the first report in this tissue, since GOM has only been described in myocardium [31].

Not all researchers consider EM as a sensitive method for CADASIL diagnosis [20], [32]. In particular, Malandrini et al. [32] reported a sensitivity as low as 57%, which they attributed to the difficulty of examining a sufficient number of arteries in skin biopsies and to the variable involvement of extracerebral vessels. Based on our experience, the sensitivity of the technique is influenced by EM operator skills in identifying GOM and in distinguishing it from similar but unrelated deposits. To avoid error it is essential to adhere strictly to current guidelines for GOM identification [11], [13]. Furthermore, examination of an adequate number of medium and small arteries is mandatory. Even though GOM was detected in the first or the first two arteries from all 13 GOM-positive patients, we agree with Markus et al. [20] that a minimum of five arteries should be examined in each patient. Based on this criterion patients with NOTCH3 mutations overlapped with GOM-positive ones.

Immunohistochemistry has been proposed by Joutel et al. [33] to be a reliable and readily available tool for CADASIL diagnosis, and to have a sensitivity of 96% and a specificity of 100%; in contrast Lesnik Oberstein et al. [34] reported a large number of false-negative and even false-positive results in a study of 62 patients.

After EM detection of GOM and negative testing for the more common NOTCH3 mutations comprehensive NOTCH3 screening should be performed. Testing only exons 3 and 4, as suggested by Kalimo et al. [35], would have identified the mutations in only 30% of our patients. These data agree with previous reports showing that mutations in exons 3 and 4 account for only 36% of Italian CADASIL patients [36] and disclosing the genetic heterogeneity of CADASIL in a small Italian subpopulation [37]. Our GOM-positive patient #6 is a case in point, because complete sequencing of the gene, 11 years after biopsy examination and negative testing for exon 3 and 4 mutations, identified a mutation in exon 10.

## Conclusions

In conclusion, our study confirms previous observations and extends our knowledge of GOM distribution in tissues where it has never been described before, such as pericardium. Moreover, we report a novel NOTCH3 mutation in exon 3, detected in three siblings. Our findings also confirm the genetic heterogeneity of CADASIL in a small Italian subpopulation, emphasizing the difficulties in designing algorithms for molecular diagnosis. Finally, our data support the notion that EM analysis is critical to highlight the need for screening NOTCH3 exons 2–24.
